# Investigating phloem transport dynamics in Arabidopsis through compartmental modelling of positron emission tomography data

**DOI:** 10.1186/s13007-026-01525-6

**Published:** 2026-03-30

**Authors:** Jens Mincke, Karen J. Kloth, Sarah Verbeke, Ken Kersemans, Stefaan Vandenberghe, Christian Vanhove, Kathy Steppe

**Affiliations:** 1 Laboratory of Plant Ecology, Department of Plants and Crops, Faculty of Bioscience Engineering, Ghent University, Ghent, Belgium; 2 MEDISIP - INFINITY - IBiTech, Department of Electronics and Information Systems, Faculty of Engineering and Architecture, Ghent University, Ghent, Belgium; 3 Laboratory of Entomology, Wageningen University & Research, Wageningen, The Netherlands; 4 Medical Molecular Imaging and Therapy, Department of Radiology and Nuclear Medicine, Ghent University Hospital, Ghent, Belgium

**Keywords:** Phloem, Arabidopsis, Positron emission tomography (PET), Carbon-11 (11C), Leakage-retrieval, Source-sink relations, Carbohydrate partitioning, Compartmental modelling, Carbon allocation

## Abstract

**Background:**

Phloem is the long-distance transport tissue of vascular plants in which photoassimilates are distributed from sources (e.g., leaves) to sinks (e.g., roots, fruits). Phloem transport occurs under pressure, making it very sensitive to manipulation and almost experimentally inaccessible. Therefore, functional data on phloem speed and dynamic distribution of photoassimilates along the transport pathway are still scarce, both in trees and herbaceous plants. This study presents a methodological pipeline to image phloem transport in very thin shoots of the model plant Arabidopsis using photosynthetic uptake of ^11^CO_2_ and state-of-the-art positron emission tomography (PET).

**Results:**

Successful application of the latest generation preclinical PET scanners allowed *in vivo* visualization of internal movement of ^11^C-labelled photoassimilates inside primary and secondary shoots of 1 to 2 mm diameter every 5 min. Using this data as input in a compartmental model enabled estimation of (i) phloem front speed, and (ii) radial carbon partitioning between leakage-retrieval phloem, carbon storage and respiratory efflux. The methodology shows that the phloem front speed of recently fixed carbon in primary shoots was almost two-fold the speed in secondary shoots (128 *vs.* 70 µm s^−1^). Furthermore, it was estimated that the fraction of recently fixed ^11^CO_2_ that was unloaded from the phloem to the surrounding storage cells and retrieved back into the phloem was higher in primary shoots than in secondary shoots, and that allocation to the storage compartment was higher in secondary shoots. Within the primary shoot, the fraction of unloading and retrieval of the ^11^C-labelled photosynthates increased towards the inflorescence.

**Conclusion:**

Here, we demonstrate the synergistic application of high-resolution PET scanning and compartmental modelling as a promising approach to advance our understanding of phloem dynamics in small-dimension plants, such as the model plant Arabidopsis. With this, an opportunity is created to explore the genetic basis of phloem dynamics.

**Supplementary Information:**

The online version contains supplementary material available at 10.1186/s13007-026-01525-6.

## Background

Phloem tissue provides the long-distance transport of photosynthates, signal molecules and other compounds between plant organs. Carbohydrate transport in phloem tissue is required to maintain proper functioning of photosynthesis in source organs and to support growth of sink tissues where assimilates are unloaded. Münch [40] described the universal concept of mass flow, applicable to different plant shapes and sizes, where a turgor gradient created by photoassimilates is the driving force of phloem transport. Later, the leakage-retrieval theory [55] was added to the bulk flow hypothesis, underlining that loading and unloading not only takes place in sources and sinks, but everywhere along the pathway in order to regulate the osmotic gradient [52]. On the organism level, three functional phloem sections can be categorized: (i) collection phloem in source tissue, where carbohydrate loading takes place, (ii) release phloem in sink tissue, where carbohydrates are unloaded, and (iii) transport phloem along the pathway between source and sink [3, 52]. Remarkably little is, however, known about the genetic basis and (intra)specific variation in phloem sap allocation [57]. This can partially be attributed to the technical challenges, since phloem tissue is a pressurized system which makes it very sensitive to manipulation [21, 44, 52, 58]. Particularly in small herbaceous plants, such as the model plant *Arabidopsis thaliana* L. (Heynh.), this has hampered scientific progress in the field of phloem biology.

Arabidopsis was originally selected as a model species for its convenient short life cycle, small size and selfing compatibility [48]. In roughly three decades, the genomes of more than thousand natural Arabidopsis accessions and a vast number of transcriptomes and transgenic lines have been generated [5, 10], making Arabidopsis an interesting study system for phloem biology. Also from an ecological perspective, Arabidopsis is of interest, as it usually pioneers in open habitats and flowers early in the season to escape interspecific competition. Winter annuals can even flower within four weeks after germination [45]. Fast and efficient allocation of photoassimilates is of importance for a species with such a short life span. Like most herbaceous plants, Arabidopsis is an apoplastic loader, relying on active transport of sugars across the plasma membranes by hexose- and sucrose-transport proteins, such as SUC and SWEET proteins [42, 60]. Other osmolytes are transported into the phloem sap by e.g., amino acid carriers, aquaporins and ion channels to maintain osmotic gradients [4]. Rapid sugar allocation to the primary inflorescence shoot is the initial regulator of apical dominance that suppresses outgrowth of secondary shoots and regulates branching architecture [35]. In Arabidopsis, the rosette is the main carbohydrate supplier for the primary inflorescence shoot and roots, while photosynthates assimilated in green parts of the inflorescence itself mostly seem to be allocated to secondary shoots [41, 53].

One of the techniques for studying phloem transport in Arabidopsis involves isotope labelling, using either radioactive or stable isotopes. Radioactive pulse-chase experiments have been performed where either whole rosettes or single leaves were exposed to short pulses of ^14^CO_2_ and, after a chase period, accumulation of ^14^C-containing sugars in different sections of the plant is determined using liquid scintillation counting [2, 14, 28, 29]. Chase periods are typically one hour but are reported from 15 min up to several hours. Also stable isotope tracing, with for example ^13^C, has been applied to study metabolic allocation of sucrose in Arabidopsis [13]. Application of radioactive and stable isotopes does, however, require invasive or destructive sampling, restricting both temporal and spatial data to discrete time points and bulk tissues, respectively. However, the combination of ^14^CO_2_ and Bremsstrahlung has been used to study *in vivo* photosynthate partitioning in small (i.e., 30-cm long) kiwifruit cuttings [7] as well as Arabidopsis plants [6]. The very long half-life of ^14^C, i.e., 5730 years, allowed long-duration experiments in these studies (i.e., 48 h and 72 h, respectively) but required a long label exposure time (i.e., 0.5 h and 2 h, respectively) compared to destructive ^14^C-detection through liquid scintillation counting (i.e., generally 5 min). Such extended exposure time may constitute a methodological limitation, as plant physiological responses can be altered within the timeframe of the labelling period. The combination of both real-time *in vivo* measurements and the short pulse labelling would yield more insights into the temporal and spatial aspects of phloem sap dynamics.

In Arabidopsis roots, phloem-mobile fluorescent probes, such as esculin and dichlorofluorescin diacetate, have mostly been used for real-time imaging [17, 25, 26]. These fluorescent probes are relatively easy to apply, quickly load into the phloem and require standard fluorescence microscopic equipment for imaging, either by tracking the movement of the fluorescent front, or the fluorescence recovery after photobleaching in the sieve tubes. These properties make them an excellent method for *in vivo* phenotyping of phloem transport on medium- to high-throughput scale. Their main limitation is, however, that they are optimized for root tissue of relatively young plants, because the vascular bundle in older above-ground tissue is confounded by autofluorescence of upper cell layers. Moreover, they do not track the carbon specifically, only the flow speed of the phloem. With magnetic resonance imaging (MRI), water volume flow rates of xylem and phloem can be assessed non-invasively at high resolution scale [27, 54]. Latest techniques in positron emission tomography (PET) now offer an alternative imaging methodology of the above-ground plant organs [20]. PET allows to follow radiolabelled molecules in 2D or 3D *in vivo* using short pulses. Specifically the latest-generation preclinical PET scanners (that have been developed for preclinical trials on small rodents) make this possible at a sub-millimeter spatial resolution [20, 30, 38]. This resolution permits, likely for the first time ever, imaging of thin structures like Arabidopsis shoots.

In this study, we present a complete methodological pipeline for non-invasive, real-time quantification of phloem front speed and radial carbon partitioning in thin Arabidopsis shoots, combining high-resolution PET imaging with a four-compartment model. To this end, we use radioactive carbon-11 (^11^C), an artificial radioisotope with a half-life of 20.4 min [20, 30, 38]. Using a compartmental model [9, 20] on the ^11^C time-activity curves obtained from four adjacent regions, both the phloem front speed and also radial carbon partitioning can be tracked. This approach enables the study of parameters related to leakage-retrieval, carbon storage and carbon efflux as part of shoot respiration. The pipeline is demonstrated on Arabidopsis shoots of 1.3-to-1.8-mm diameter. Using this methodology, we explored differences in phloem front speed and radial carbon partitioning between primary and secondary shoots. Furthermore, the effect of shoot height on carbon allocation parameters related to leakage-retrieval, carbon storage and respiratory efflux was investigated. With that, we present new insights and a novel approach that relies on the concerted effort of high-resolution PET scanning and compartmental modelling. These two techniques, when combined, open up a suite of new possibilities to test theoretical frameworks of phloem transport and to address biological questions related to the underlying mechanisms of carbohydrate partitioning.

## Methods

### Plant material

Arabidopsis Col-0 accession (CS60000) seeds were cold stratified for 72 h at 4 °C and subsequently sown into 6-cm-diameter pots with Arabidopsis potting soil (Lentse Potgrond). Plants were grown in a climate room at 26 ± 1 °C during the day and 23 ± 0.5 °C during the night, 50 to 70% relative humidity (RH), and an 8:16 light (L): dark (D) photoperiod. Plants were treated with Entonem (Koppert Biological Systems) once a week to avoid sciarid flies. Plants started to flower after approximately 8.5 weeks. For PET scanning (see below) plants were transported to INFINITY lab, i.e., the pre-clinical core imaging facility of Ghent University, where they were kept at 20 °C, while PPFD of 295 µmol m^−2^ s^−1^ was supplied via a white LED bar (Lucis, Parus Europe, The Netherlands). Four plants were scanned at the age of 9 to 10 weeks. All plants had either four or five secondary shoots.

### Production and formulation of ^11^CO_2_

Radioactive ^11^CO_2_ was produced by the cyclotron (18 MeV protons, IBA, Belgium) of Ghent University Hospital. Inside the cyclotron a N_2_/H_2_ (5%) target was bombarded with a highly accelerated proton (i.e., H^+^) causing a (p, α) nuclear reaction, which resulted in the formation of ^11^CH_4_. This [^11^C]methane was subsequently oxidized via cobalt oxide to yield ^11^CO_2_ as described by Landais & Finn [32]. By bubbling the ^11^CO_2_ gas through a 1 M solution of NaOH it was captured and dissolved as Na_2_^11^CO_3_ in the high-pH solution. A syringe was drawn from this NaOH solution with dissolved ^11^CO_2_ for convenient transport to the PET room. The mean (± SE) starting activity to which plants were exposed was 88.4 ± 9.2 MBq.

### Experimental set-up, ^11^CO_2_-labelling, and positron emission tomography data collection

**Fig. 1:**
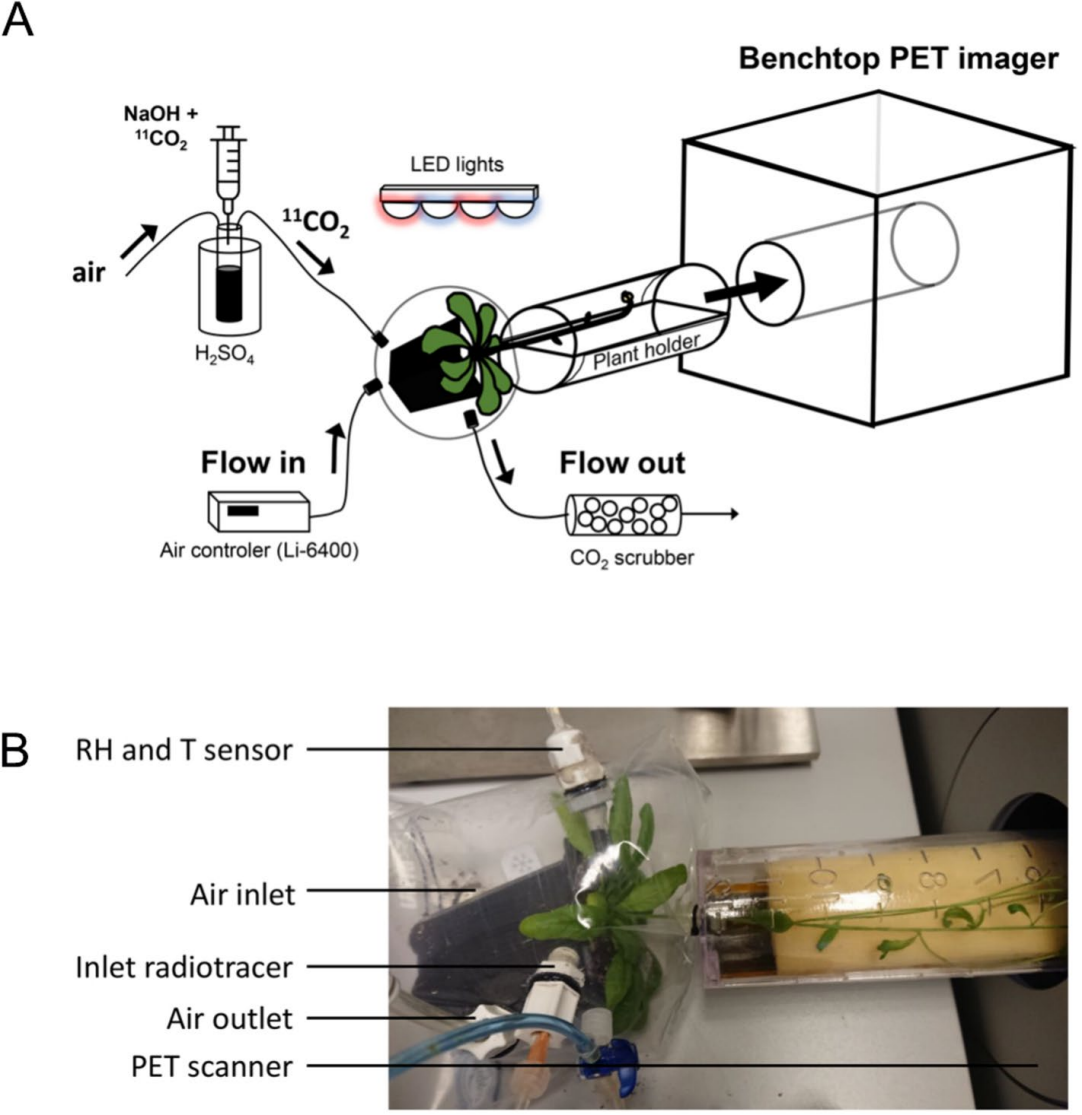
Positron emission tomography (PET) scan platform applied for measuring phloem transport dynamics in Arabidopsis inflorescences based on ^11^CO_2_ labelling. **A** Schematic configuration of the platform. **B** Arabidopsis plant with rosette in an airtight labelling bag and the inflorescence laying on the PET bed that is mounted for PET detection. Abbreviations: RH: relative humidity, T: temperature, LED: light-emitting diodes.

Safe administration of ^11^CO_2_ to Arabidopsis leaves was ensured by enclosing the rosette in a labelling bag cuvette (zipper bag with volume of 1 L) that was hermetically sealed around the primary shoot (Fig. 1). This was realised at the base of the primary shoot using lubricated hard (inner) and soft (outer) concentric cylindrical tubing in combination with straps to tighten the labelling bag [38]. Caution was made to prevent damaging the shoot as the phloem tissue is located at the transaxial periphery of the shoot. The labelling bag was connected to two tubes passing air continuously to and from the rosette. A portable gas exchange system (model Li-6400, Li-Cor Inc., Lincoln, NE, USA) (Fig. 1) supplied air containing 400 ppm CO_2_. Via a third tube, connected to the labelling bag cuvette, gaseous ^11^CO_2_ was administered to the rosette. This tube was connected to the labelling bag cuvette by a needle inserted through a septum (Fig. 1). A syringe containing the dissolved ^11^CO_2_ was emptied through a needle in a sealed vial containing an excess of an acidic solution (H_2_SO_4_ in water). The acid–base neutralisation triggered the release of ^11^CO_2_ from the solution. Using a 60-mL syringe, air was blown into the solution (through a second needle that was submerged in the solution) to direct the released ^11^CO_2_ (accumulating in the headspace of the vial) through a third wider needle positioned in the headspace of the vial. This needle was connected to the small tube (to reduce dead-volume) supplying ^11^CO_2_ to the labelling bag cuvette. Just before the basic solution was added to the acid, the air flow to the rosette was stopped for seven minutes to ensure sufficient uptake of ^11^CO_2_ by the leaves. Subsequently, the 60-mL syringe (filled with air) was gently deflated inside the vial so that the rosette leaves were pulse-labelled with ^11^CO_2_.

To prevent radioactivity from escaping into the atmosphere, the air flowing out of the labelling bag cuvette was directed to a CO_2_ scrubbing column containing soda lime pellets (calcium hydroxide on sodium carbonate carrier, Merck, Overijse, Belgium) (Fig. 1A).

Plants were enclosed in the labelling bag cuvette and placed horizontally at least 1 h before radioactive labelling. During this period and during the entire scan, a light source (ten red and blue LED lights, GreenPower LED strings 0842 LF Red and WPO 83 LF Blue, Philips, the Netherlands) provided about 200 μmol photons m^−2^ s^−1^ photosynthetic active radiation (PAR) to the rosette leaves.

Once ^11^CO_2_ was administered, phloem transport dynamics in intact Arabidopsis shoots were imaged using a preclinical benchtop β-CUBE PET scanner (MOLECUBES, a Bruker Company, Ghent, Belgium) (Fig. 1). The PET scanner is characterised by a small ring of detectors (inner diameter and depth of 7.8 and 13.0 cm, respectively) defining the field of view (FOV) which acquired radioactive signals for 2 h starting from the exposure of the rosette leaves to gaseous ^11^CO_2_. After scanning, dynamic reconstruction of the registered data was done by the reconstruction software (MOLECUBES, a Bruker Company, Ghent, Belgium) using the 3-D ordered-subsets expectation maximization (OSEM) algorithm, with 50 iterations, 4 subsets and 5-min time frames. The result is a decay-corrected 4D image (x, y, z, t) consisting of 24 timeframes with 192 × 192 × 384 voxels with 400 µm isotropic voxel size.

### Image analysis of positron emission tomography data to construct time-activity curves

**Fig. 2:**
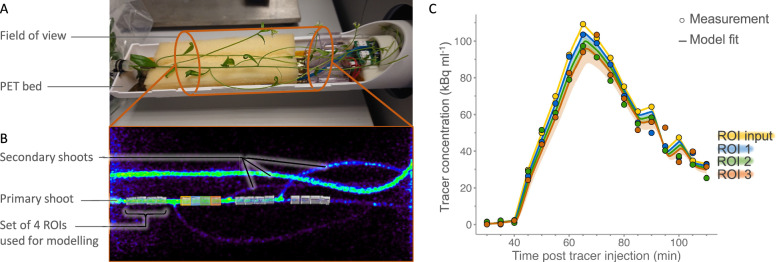
**A** Picture of an Arabidopsis plant in the positron emission tomography (PET) bed without the transparent bed cover (shown in Fig. 1B) along with the cylindrical field of view (FOV) region in which radioactivity was detected by the PET scanner. **B** Corresponding static rendered PET image of the FOV showing the primary and several secondary shoots. The static PET image shows the cumulative activity that was recorded during the entire scan time (i.e., 120 min). PET image analysis involves drawing sets of four consecutive regions of interest (ROIs) of which the input is used for modelling. Four ROI sets, each containing four ROIs, are drawn on the primary shoot. Because not all secondary shoots are (completely) visible on the PET image, ROI sets are drawn on at least two different secondary shoots per plant to obtain an equal total number of four ROI sets per plant. ROI sets on secondary shoots are not shown in panel (**B**). By extracting tracer concentrations within a set of four consecutive ROIs of the corresponding dynamic PET image (i.e., 5 min time frames) time-activity curves (TACs) are obtained. **C** Example of TACs (circles) of the second ROI set shown in panel (**B**). Time is expressed in minutes after pulse-labelling gaseous ^11^CO_2_ to the rosette of the Arabidopsis plant. A compartmental model, representing the system under study, is then fitted to the measured TACs resulting in continuous lines. 95% confidence interval of each simulation is represented by a shaded area.

The 4D images were imported in the open-source software tool Amide [34] for visualisation and image analysis. Moreover, cylinder-shaped regions of interest (ROIs) were drawn around shoot tissues along the direction of phloem flow. Sets of four consecutive ROIs (input ROI, ROIs 1–3, Fig. 2B) were drawn on the PET images. ROI size represents a balance between signal-to-noise ratio and physiological resolution. ROIs that are too small yield noisy time-activity curves (TACs), which impedes stable model fitting. Conversely, excessively large ROIs average tracer dynamics over longer stem segments, reducing physiological relevance because phloem transport properties can vary along the shoot, whereas the model assumes constant phloem front speed in the consecutive ROIs. ROIs drawn on the primary shoot had a length and diameter of 4 and 3 mm, respectively. Since such ROIs drawn on secondary shoots resulted in noisy data, the dimensions of a set of four secondary shoot ROIs were enlarged to a length and diameter of 10 and 5 mm per ROI. Subsequently, the average concentration of recently fixed ^11^C-tracer accumulating in each ROI was calculated per 5 min timeframes (in MBq mL^−1^) in the reconstruction software (MOLECUBES, a Bruker Company, Ghent, Belgium). As reconstructed images were corrected for decay, so were the resulting time-activity curves (Fig. 2C) that were exported as text files. For each of the four Arabidopsis plants, four ROI sets (each containing four consecutive ROIs) were drawn on the primary shoot (n = 16). Because not all secondary shoots were (completely) visible on the PET image, ROI sets (again each containing four consecutive ROIs) were drawn on at least two different secondary shoots per plant to obtain an equal total number of ROI sets (n = 16).

A typical TAC follows a pulse signal (example in Fig. 2C). Each distally located ROI shows a lower concentration with respect to the upstream adjacent ROI which is especially visible at the maximum of the signal. This can be linked to local storage of tracer in the upstream ROI and/or tracer efflux to the atmosphere, which is the base for compartmental modelling.

### Compartmental model development

**Fig. 3:**
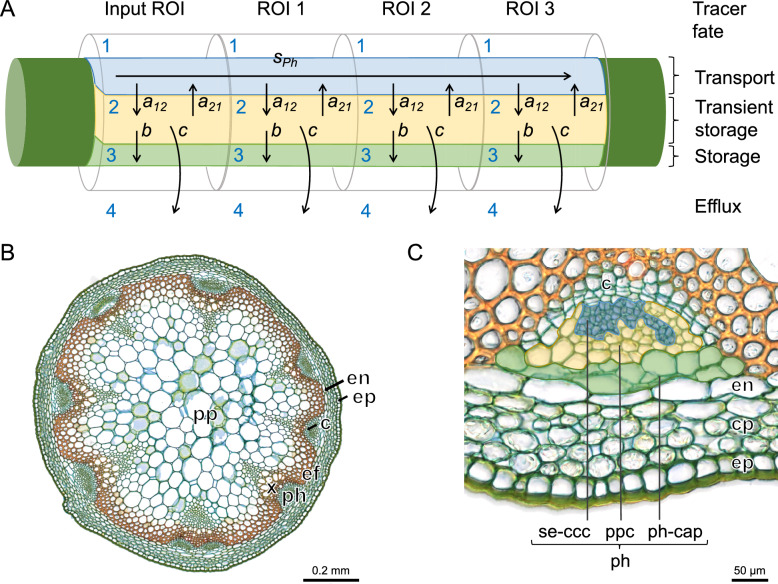
**A** Schematic representation of the four-compartment model used to simulate tracer dynamics in the defined regions of interest (ROIs) along the Arabidopsis primary and secondary shoots. Tracer that enters a ROI is defined by phloem speed *s*_*Ph*_ (µm s^−1^) and exchange parameters *a*_*12*_, *a*_*21*_, *b* and *c* (s^−1^). Parameter *s*_*Ph*_ describes the phloem front speed of tracer particles in compartment 1. From the phloem conduits a fraction *a*_*12*_ of the tracer will move to the transient storage compartment 2, from which (i) a fraction *a*_*21*_ can be retrieved to the phloem conduits, (ii) a fraction *b* can enter storage compartment 3, where it is immobilized and stored, or (iii) a fraction *c* can exit to the atmosphere (i.e., compartment 4 outside the ROI) through respiratory efflux. Parameters are assumed equal in each ROI, and data for the input ROI data is directly derived from measured activity. **B** Anatomical cross-section and **C** detail of Arabidopsis primary shoot. For histological preparation, shoot segments (from plants different from those imaged) were glued to a vibratome stage using superglue (Roticoll, Carl Roth). Cross-sections of 25 µm thickness, prepared with a vibrating microtome (HM 650 V, ThermoScientific, Germany), were stained with 0.5% w/v astrablue, 0.5% w/v chrysoidine and 0.5% w/v acridine red for three minutes, dehydrated in isopropyl alcohol and mounted in euparal (Carl Roth). Slides were observed with a Nikon Ni-U light microscope and images were recorded using a Nikon DS-Fi3 camera. pp: pith parenchyma, en: endodermis, ep: epidermis + cuticle, c: cambium, ef: extraxylary fibers, ph: phloem, x: xylem, cp: cortex parenchyma, se-ccc: sieve element-companion cell complex (blue cell group in panel **C**) ppc: phloem parenchyma cells (yellow cell group in **C**), ph-cap: phloem cap, with combination of S-cells and M-cells (green cell group in panel **C**). S-cells store glucosinolates in the vacuole, whereas M-cells have myrosinase activity, which hydrolyses glucosinolates. There are only a few M-cells per phloem cap with each of them located on the side. Cell groups indicated in panel (**C**) are approximations since labelling each cell in the phloem is not straightforward. In general, ppc are larger than cells of se-ccc. Note that se-ccc, ppc and s-cells indicated in panel (**C**) correspond to compartments 1, 2 and 3, respectively, of panel (**A**).

The acquired TACs were used as input for model calibration of a four-compartment model that was implemented and calibrated in the plant modelling software PhytoSim (Plant AnalytiX, Mariakerke, Belgium). The model (Fig. 3) describes tracer transport, exchange, and loss within four compartments using transport and exchange parameters, based on Mincke et al. [37], Hubeau et al. [19] and the mechanistic model of Bühler et al. [9]. The tracer concentration measured in each ROI represents the integrated response of all different processes happening in that ROI that affect this tracer. Compartmental modelling aimed to disentangle these processes by dividing each ROI into four compartments that each represent a different space (i.e. three cell types and the atmosphere) where the tracer might end up due to interactions between them. The four-compartment model specifically calculates tracer concentration (*T*_*C*_ in MBq ml^−1^) within a ROI as the sum of the concentration of its four compartments (Fig. 3C): (i) phloem tissue conduits in which tracer is transported (compartment 1: sieve element-companion cell complexes), (ii) a transient storage compartment (compartment 2: phloem parenchyma) that exchanges tracer with the local transport pathway and the other two compartments, (iii) a storage compartment where tracer becomes immobilized (compartment 3: phloem cap), and (iv) the atmosphere in which tracer ends up through efflux.

The change in tracer concentration of compartments 1–3 is described by

1 $$\frac{d{{T}_{C}}_{i}^{1}}{dt}= \frac{{s}_{Ph}}{l}\cdot {{T}_{C}}_{i-1}^{1} - \frac{{s}_{Ph}}{l}\cdot {{T}_{C}}_{i}^{1} - {a}_{12}\cdot {{T}_{C}}_{i}^{1}+h \cdot {a}_{12}\cdot {{T}_{C}}_{i}^{2}$$

2 $$\frac{d{{T}_{C}}_{i}^{2}}{dt} = {a}_{12}\cdot {{T}_{C}}_{i}^{1} -h {\cdot a}_{12}\cdot {{T}_{C}}_{i}^{2}- b\cdot {{T}_{C}}_{i}^{2} - c\cdot {{T}_{C}}_{i}^{2}$$

3 $$\frac{d{{T}_{C}}_{i}^{3}}{dt} = b\cdot {{T}_{C}}_{i}^{2}$$

where superscript denotes sub-compartment number and subscript *i* the ROI number. Constant *l* is the ROI length (i.e., 4 and 10 mm for primary and secondary shoots, respectively). The model parameters are the phloem front speed *s*_*Ph*_ of tracer particles in the phloem tissue [µm s^−1^], loss and regain of tracer fraction rates *a*_*12*_ [s^−1^] and *a*_*21*_ [s^−1^] by steady unloading and reloading, respectively, from and to the transport pathway, respectively, and exchange fraction rates *b* [s^−1^] and *c* [s^−1^] representing the loss of tracer to the storage compartment and the atmosphere over time, respectively. In the storage compartment, the tracer is immobilized and movement or exchange with other compartments is no longer possible. The loss of tracer to the atmosphere is linked to respiratory activity, which produces gaseous CO_2_ that effluxes to the atmosphere.

Note that parameter *s*_*Ph*_ denotes the phloem front speed of the fastest particles, i.e., the particles in compartment (1) which might be distinctively different from the group speed. In correspondence to the model described by Bühler et al. [9], we assumed a single effective speed *s*_*Ph*_ (corresponding to ‘plug flow’ or idealized flow without any mixing of fluid particles) per ROI set because this allows neglecting compartment sizes and shapes as well as additional obstacles (e.g., sieve plates). Parameters* a*_*12*_, *b* and *c* as well as the calculated parameter *a*_*21*_*,* represent fractions of the total tracer concentration within a ROI flowing between compartments over time and thus range from 0 to 1 per s. To prevent over-parameterisation of the model, exchange parameters *a*_*12*_ and *a*_*21*_ were coupled by using a fixed ratio between them. This is described by *a*_*21*_ = *h* × *a*_*12*_, where *h* is a model constant, similar to Bühler et al. [9]. Estimates of tracer transport properties, i.e., model parameters {*s*_*Ph*_, *a*_*12*_, *a*_*21*_, *b* and *c*}, were obtained by fitting the numerical solution of the partial differential model equations (model fit in Fig. 2C) to the measured recently fixed ^11^C-tracer profiles (TACs, i.e., discrete PET data in Fig. 2C). A value for *h* was obtained by first calibrating a five-parameter model {*s*_*Ph*_, *a*_*12*_, *b*, *c* and *h*} for each of the primary and secondary shoot ROI sets (n = 32). In ten cases, parameter *h* was indicated as identifiable parameter, from which the average value was calculated. Next, parameter calibration of the identifiable four-parameter model {*s*_*Ph*_, *a*_*12*_, *b* and *c*} was done for each of the 32 ROI sets where *h* was defined as a constant that equalled 0.68759 (average of ten calibrated values). Since plant dimensions did not change within one ROI set, all other parameters per set of ROIs are assumed to be constant during the PET measurement (i.e., 2 h).

The input ROI is most proximally located to the rosette compared to the other ROIs and does not receive any tracer from any ROI. In reality, tracer will enter this ROI, so values for the input ROI were directly derived from the total concentration of tracer measured in the input ROI (${T}_{{C}_{total}}$) for each time step according to

4 $${{T}_{C}}_{input}^{1}={{T}_{C}}_{total}-{{T}_{C}}_{input}^{2}-{{T}_{C}}_{input}^{3}$$

The change in tracer concentrations for the other two compartments was calculated according to Eqs. (2–3) with *i* = *input*. For the first timeframe (5 min duration), all measured tracer was assumed to be present in compartment 1 of the input ROI. We tested this assumption and found the parameter outcome to be insensitive to whether initial activity was allocated to compartment 1 only or distributed over all compartments.

### Model calibration and parameter sensitivity and identifiability analysis

Unique values for the parameter set were obtained through model calibration using a shuffled complex evolution [15], with nine complexes (i.e., two times number of model parameters + 1), which was found to result in a better overall calibration performance as described by Duan et al. [16] and an accuracy of 10^−5^, for 4500 evaluations. Using the resulting model parameters, continuous data were simulated using an adaptive step size fourth order Runge–Kutta solver (accuracy 10^−5^, maximum step size 0.01 min) [31, 43, 49]. Calibration was completed when the difference between simulated and measured TACs (root mean square error) was minimized.

Model sensitivity and identifiability analysis were assessed according to De Pauw et al. [43] by making use of the corresponding modules in the plant modelling software PhytoSim (Plant AnalytiX, Mariakerke, Belgium). The model parameter sets {*s*_*Ph*_, *a*_*12*_, *b, c*} for each calibration were found identifiable with a collinearity index that was generally between five and ten, which is below the threshold of 15. When the collinearity index value is higher than 15, the parameter subset is said to be unidentifiable [8]. Sensitivity analysis indicated that the model parameters were characterized by a high sensitivity for the model output.

### Statistics

The model output provided five transport‑related parameters (*s*_*Ph*_, *a*_*12*_, *a*_*21*_, *b*, and *c*), which served as the metrics for statistical analysis. Statistical comparisons were designed to (i) test whether these transport metrics differed between primary and secondary shoots, and (ii) evaluate whether shoot height influenced any of the parameter values.

The distribution of each of the model parameters was tested for normality with a Shapiro–Wilk test. For normally distributed data (parameters *s*_*Ph*_ and *b*), differences between primary and secondary shoots were tested using a linear mixed model via the R package nlme [46], other data distributions were selected with the fitdistrplus R package [12] and were tested with a generalized linear mixed model with the best fitting distribution (gamma distribution for *a*_*12*_, *a*_*21*_ and *c*) via the *glm* function of R, version 4.0.4 [47] with shoot type as fixed effect. As multiple measurements (e.g., four ROI sets on the primary shoot) were obtained for each of the four Arabidopsis plant, we corrected for the effect of plant individual by including it as a random effect. All models were tested against a null model with a likelihood ratio test via the lmtest package [61]. For parameter *c* no model was found to be better than the null model (Additional file 1). Effects of shoot height on transport parameters were tested via a Pearson correlation test (for normally distributed data) or a Spearman correlation test (for data without a normal distribution). Plots were made with the package ggplot2 [59].

## Results

### Image analysis and time activity curves of phloem-transported ^11^C-labelled photoassimilates

**Fig. 4:**
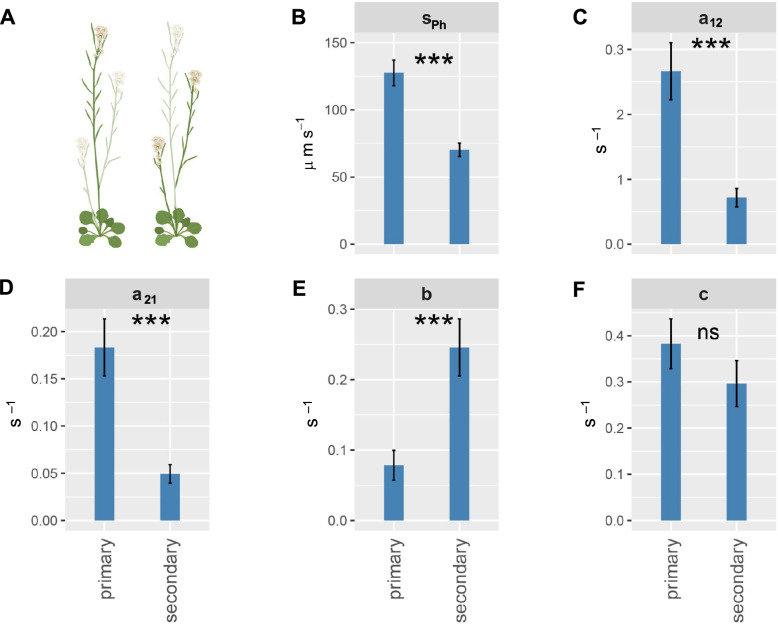
Recently fixed ^11^C-carbon allocation patterns in Arabidopsis inflorescences as estimated by compartmental modelling in **A** the primary (left) and secondary (right) shoots (non-transparent organs). **B** phloem front speed *s*_*Ph*_, the exchange rate of the carbon fraction that is **C** unloaded (*a*_*12*_), and **D** reloaded into the transport compartment (derived fraction *a*_*21*_), **E** allocated to the storage comportment (*b*), or **F** exits the plant (*c*) (bars depict mean, error bars depict standard error, *** P < 0.001, ns = not significant, (generalized) linear mixed models).

High-resolution PET imaging was successfully applied to acquire images of thin plant structures, including both primary and secondary Arabidopsis shoots. Additionally, a pipeline was successfully developed in which the understudied dynamics of phloem transport in this model plant can now be studied. Compartmental modelling revealed that recently fixed ^11^C-labeled photoassimilates are transported in sieve tubes of both primary and secondary Arabidopsis shoots with a maximum phloem front speed that varied from 95 µm s^−1^ in the secondary shoot, to 216 µm s^−1^ in the primary shoot. Overall, the phloem front speed *s*_*Ph*_ in primary shoots was almost two-fold higher compared to secondary shoots, i.e., 127.6 ± 9.5 *vs.* 70.3 ± 5.0 µm s^−1^, respectively (mean ± standard error, Fig. 4B, P < 0.001, linear mixed model, Additional file 1). Furthermore, the compartmental model identified and quantified the exchange fraction rate parameters *a*_*12*_ (i.e., rate of tracer fraction exchange, supposedly going from phloem conduits to the companion cells, surrounding phloem parenchyma and apoplast), *a*_21_ (i.e., rate of tracer fraction exchange, supposedly from companion cells and phloem parenchyma that is retrieved in the sieve tubes), *b* (i.e., the rate of tracer fraction exchange supposedly within the phloem parenchyma and apoplast that gets stored in the phloem cap) and *c* (i.e., rate at which the tracer diffuses from supposedly phloem parenchyma to the atmosphere via efflux, expressed as a fraction). The sensitivity analysis showed that parameters were highly sensitive for the model output and some interdependent. No correlations were found, except for *a*_*12*_ and *b* (P = 0.02, r = 0.58) (for more details on identifiability and sensitivity analyses, see Materials and Methods).

### Tracer detection in small-diameter shoots

To assess if PET is suitable for measurements in shoots with a stem diameter below 2 mm, additional calculations were performed. An unavoidable characteristic of PET imaging is the positron range, which is the small distance a positron travels when losing its kinetic energy between the point of creation (i.e., decay of ^11^C atom) and annihilation (i.e., conversion to gamma rays). The mean positron range is different for radionuclides and is 1.2 mm for ^11^C [11]. This is close to the diameter of Arabidopsis shoots making it questionable if positrons originating within such thin shoots actually annihilate in it or escape and travel in the air to annihilate onto the surface of another tissue. To address this question, the average travel distance was determined for positrons that originate in phloem tissue of cylindrical Arabidopsis shoots with diameter *d* (Additional file 2). Hereby, the center of the phloem tissue was assumed to be located at 0.09 × *d* of the stem surface (Fig. S1) as determined by transverse Arabidopsis sections. The ^11^C-positron range can increase up to maximum 4.2 mm [11] so that the positron travel distance within the stem was limited to 4.2 mm. The average travel distance was then converted to the annihilation probability (*P*_*annihilation*_) according to Jodal et al. [22]. The Arabidopsis shoots that were imaged in this study had a diameter between 1.3 and 1.8 mm which corresponds to *P*_*annihilation*_ of 67 to 76% (Additional file 2). Even though phloem tissue is located close to the surface of the shoot, it was calculated that most of the positrons still annihilate within the shoot, which assures that acquired PET images are the result of actual phloem transport dynamics and not related to random annihilation of escaped positrons onto the shoot surface.

### Differences in partitioning between primary and secondary shoots

Phloem transport was studied in the primary and secondary shoots of flowering Col-0 plants in a region from 11 to 21 cm above the rosette in four discrete locations on the shoot with a set of four ROIs in each location (Fig. 2B). As no flowers or siliques were present in the FOV, the observed area involved the conduits between source leaves and reproductive sink organs, referred to as transport phloem [3]. Each of the model-derived parameters *a*_*12*_, *b* and *c*, provide an indication on how the recently fixed carbon got fractionated into different phloem compartments during the 2-h recording. Hereby, the majority of recently fixed ^11^C-carbon was represented in phloem transport, followed by an unloaded fraction rate (*a*_*12*_) that was either retrieved for transport (*a*_*21*_) or allocated to the ‘storage’ compartment (*b*) or left the plant via efflux (*c*). Compartmental modelling of the carbon fluxes indicated that partitioning of recently fixed ^11^C-carbon differed between primary and secondary shoots. Parameter *a*_*12*_ (and the thereof derived parameter *a*_*21*_) was significantly higher in the primary shoot compared to secondary shoots, indicative of a higher ^11^C-carbon fraction that is unloaded (*a*_*12*_: 0.27 ± 0.04 s^−1^ *vs.* 0.07 ± 0.01 s^−1^ (mean ± standard error), P < 0.001, generalized linear mixed model, Fig. 4C, Additional file 1) and retrieved (*a*_*21*_: 0.18 ± 0.03 s^−1^ *vs.* 0.05 ± 0.01 s^−1^, P < 0.001, generalized linear mixed model, Fig. 4D). The exchange fraction rate of unloaded ^11^C-labeled photosynthates allocated to storage compartment 3 was higher in secondary shoots (*b*: 0.08 ± 0.02 s^−1^ vs. 0.25 ± 0.04 s^−1^, P < 0.001, linear mixed model, Fig. 4E), although the exchange fraction rate of unloaded ^11^C-labeled photosynthates to efflux (presumably via respiration) was comparable between shoot types (*c*: 0.38 ± 0.05 s^−1^ *vs.* 0.30 ± 0.05 s^−1^, P = 0.26, generalized linear mixed model, Fig. 4F). Overall, it can be summarized that the compartmental model indicated that unloading and retrieval are stronger components in the primary shoots, while storage is relatively stronger in secondary shoots.

### Effect of shoot height on tracer transport

**Fig. 5:**
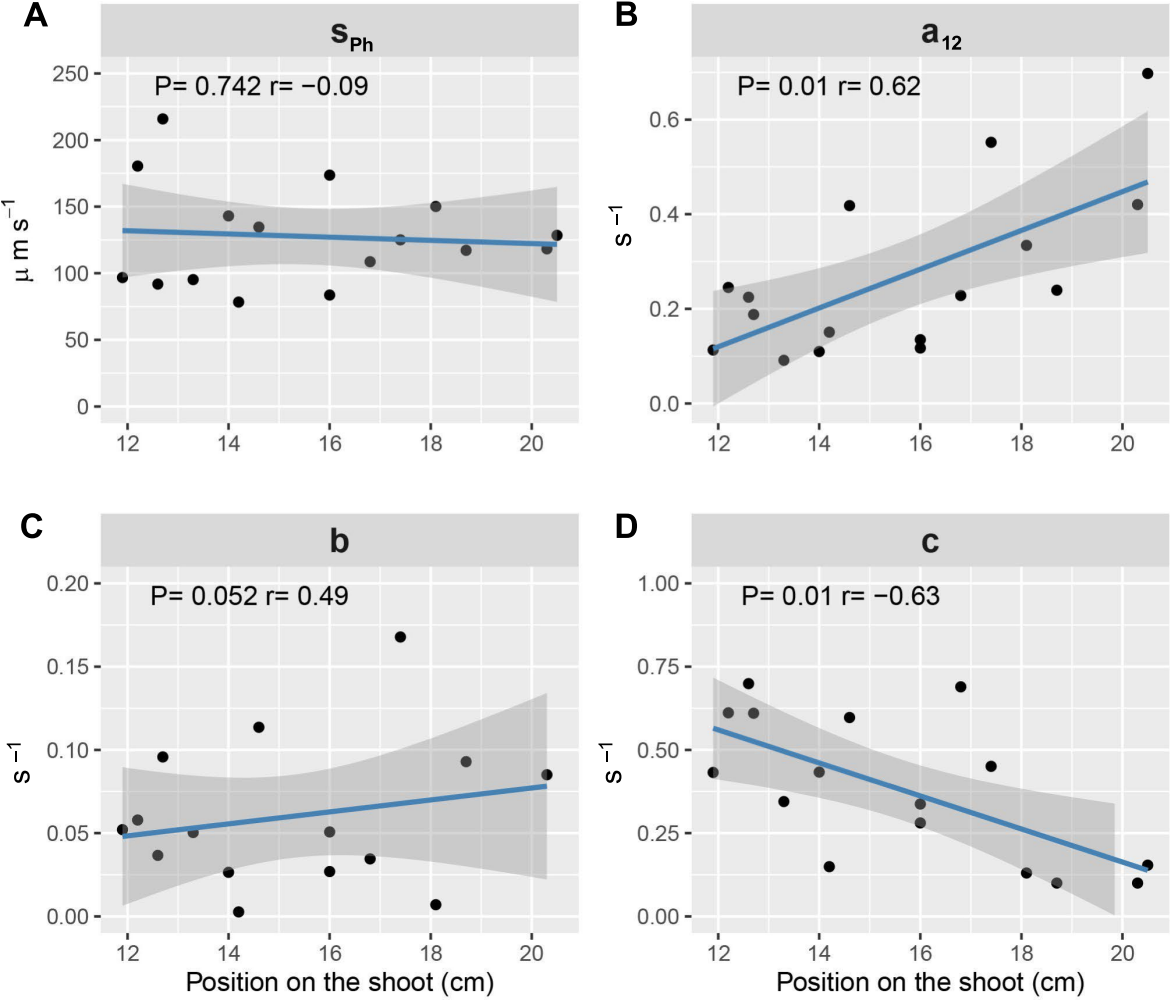
Correlations between recently fixed ^11^C-carbon transport speed/ partitioning and position along the primary Arabidopsis shoot. **A** Phloem front speed of carbon transport (*s*_*Ph*_), **B** rate at which recently fixed ^11^C-carbon fraction is unloaded (*a*_*12*_), **C** rate at which the unloaded fraction is allocated to the storage comportment (*b*), **D** rate at which the unloaded fraction exits the plant (*c*) (position on the shoot is measured from the rosette (0 cm) upwards; correlations with *a*_*12*_ and *c* have been tested with a Spearman correlation test, *s*_*Ph*_ and *b* with a Pearson correlation test).

With TACs obtained on four different heights along each 10-cm long FOV, recently fixed ^11^C-carbon dynamics could be studied on the vertical axis of the primary inflorescence shoots. Phloem front speed was unaffected by shoot height and showed less variation between individual plants in higher sections of the stem, where it stabilized around 125 µm s^−1^ (Fig. 5A). Compartmental modelling revealed that the unloaded fraction rate increased along the shoot axis (*a*_*12*_) from 0.19 ± 0.04 s^−1^ in the lower half of the FOV to 0.34 ± 0.07 s^−1^ in the upper half (mean ± standard error, Fig. 5B). The estimated retrieval fraction rate (*a*_*21*_) followed the same increase over the shoot axis, as it is the product of *a*_*12*_ and the constant *h*. The exchange rate of fraction *b* allocated to storage did not show a significant trend (Fig. 5C), but the exchange rate of the fraction of unloaded ^11^C-labelled photoassimilates destined for efflux (*c*) decreased with stem height (Fig. 5D). Compartmental modelling hence indicated that higher in the stem relatively more recently fixed ^11^C-carbon is used for unloading, retrieval and less for CO_2_ efflux.

## Discussion

### Feasibility of ^11^C‑labeling and PET in thin Arabidopsis stems to measure phloem dynamics

To obtain physiologically relevant data about phloem transport functioning, the most important criterion is that the plant under study is treated in the least invasive way possible. Yet, simultaneously, a detectable substance needs to be present in phloem conduits. Autofluorescence in older, above-ground tissue limits the use of fluorescent probes for this purpose. Non-invasive radiotracer assays using ^14^CO₂ have long been used to study transport of photoassimilates in phloem, but generally result in dynamic data with a low temporal resolution [2, 28, 29]. This can be related to the difficulty in detection of the emitted low-energy beta particles (i.e. electrons or β^–^) which requires destructive procedures to have close contact with sample (i.e. liquid scintillation detection). This methodological challenge can be circumvented by detecting the Bremsstrahlung x-rays that beta particles emit when slowing down upon interacting with matter. In this way ^14^C-carbon can be detected from the outside of the sample. However, with this method, phloem transport can only be visualized (i) in very thin structures like leaves and fine roots [18, 36, 39] because the mean path length of a ^14^C-emitted beta particle is only 0.06 mm, or (ii) by applying high-dose labelling to allow dynamic detection [6] because ^14^C is characterized by a very long half-life of 5730 years. In contrast, labelling experiments making use of ^11^C are characterized by opposing experimental conditions because this radioactive carbon isotope has a relatively short half-life of 20.4 min and emits a high-energy positron (β^+^) particle. In combination with technical improvements in PET and the introduction of monolithic PET detection crystals, ^11^C-PET imaging at sub-millimeter resolution has become feasible [24, 51]. Monolithic PET detector crystals are characterized by a high energy resolution and sub-millimeter spatial resolution which allows, probably for the first time, dynamic imaging of thin structures, like Arabidopsis shoots, using only small amounts of radioactivity. The PET scanner used in this study makes use of monolithic crystals and has a spatial resolution of 0.8 mm, which outcompetes the traditional resolution of 1–2 mm obtained with other commercial small animal PET systems [30]. The superior spatial resolution is required to reduce partial volume effect (PVE) which can be defined as the loss of apparent activity in a small object due to a limited spatial resolution of the imaging system [50]. PVE leads to an underestimation of the activity concentration in thin objects like e.g., leaves. It was found that PVE underestimated activity concentrations in leaf tissue of *Nicotiana tabacum* by a factor 10 to 15 [1]. In order not to exclude PVE, the size of a structure must be at least twice the full width at half maximum (FWHM) resolution in x-, y- and z- dimension of the imaging system, which is the case here (1.6 mm stem diameter vs. 0.8 mm FWHM spatial resolution). The PET-scanner was hence able to better visualize phloem-transported ^11^C-labelled photoassimilates in the studied Arabidopsis shoots compared to older generation preclinical PET scanners, making isotope tracing studies feasible and trustworthy. To address whether positrons originating in near-surface phloem escape before annihilation, we calculated the within-stem annihilation probability for stems of 1.3–1.8 mm diameter. The resulting 67–76% range indicates that most positrons annihilate within the shoot, confirming that the PET signal primarily reflects actual phloem dynamics rather than random surface annihilations from escaped positrons.

Additional issues arise when comparing PET data of Arabidopsis shoots with different diameters. In thinner shoots not only the number of annihilating positrons is reduced but also the phloem area is smaller compared to thicker shoots. It might be reasonable to correct PET data (i.e., TACs) for both issues upon compartmental modelling. The reduced number of annihilating positrons can be corrected by calculating the average diameter of each ROI set and resulting *P*_*annihilation*_. To account for the phloem area, cross-sectional plant sections can be taken at different shoot heights of primary and secondary shoots of the plants. From plants of similar developmental stage as the Arabidopsis plants used for the current PET study, it was found that relative phloem area (ratio of phloem area to total area) in different sections along the inflorescence in Arabidopsis plants was more or less constant and averaged (± SE) 5.4 ± 0.3% in primary shoots and 4.6 ± 0.3% in secondary shoots. Linear correction can be used to take this issue into account. However, multiplication of TACs with a correction factor does not change the relative positioning of TACs. Hence, compartmental modelling resulted in the same parameter values of {*s*_*Ph*_,* a*_*12*_, *b*, *c*} when using both uncorrected and corrected TACs. This led us to conclude that correction of TACs is not needed when used for compartmental modelling.

Recorded activity is however reduced in thinner shoots (e.g., secondary shoots) making the data more susceptible to noise. Increasing the ROI length decreased the noise. Therefore, this issue was overcome by drawing longer ROIs on secondary shoots compared to primary shoots (ROI length of 4 *vs.* 10 mm). Since the ROI length is incorporated into the model (constant *l* in Eq. (1)) the parameter values are adapted accordingly upon model calibration.

There may, however, still be some challenges to overcome that could facilitate future PET scanning of Arabidopsis plants, which include harmonizing the environmental conditions that are prevalent in the PET room to sustain plant functioning, lack of lighting on the inflorescence during the 2-h PET acquisition, and the horizontal scanning position [38]. Whereas the first two challenges can be overcome by technical solutions such as application of a cuvette containing LED lighting, the horizontal scanning position may affect plant function in the long-term. The ^11^CO_2_-based study on maize of Karve et al. [23] where little or no effect of horizontal positioning was observed in terms of photoassimilate transport speed, ^11^C-carbon fixation, or photosynthetic CO_2_ exchange rates (measured with an infrared gas analyzer) compared to vertically positioned plants within a 3 h time frame, led us to assume that horizontal positioning in the PET scanner had also little impact on the phloem functioning of our Arabidopsis plants. Sealing of the rosette into the labelling bag remains a risky task where any disturbance due to canting and touching should be limited to a feasible minimum. Our study is, therefore, a proof of principle that with these new techniques phloem transport dynamics in small-diameter organs can be studied and PET scanning may become as such part of dedicated plant phenotyping platforms of the future.

### Compartmental modelling to identify and quantify phloem transport dynamics

An important consideration is that PET imaging and mathematical modelling go hand in hand. Since PET data in this study was collected with a spatial resolution in the order of 1 mm, it is impossible to assign from which specific anatomical tissue the signal originated. Yet, the compartmental model applied in this study could describe how fast the recently fixed ^11^C-tracer was transported in both primary and secondary Arabidopsis shoots, and enabled quantification of the fraction of tracer that is dynamically distributed over different compartments, associated with different phloem cell types (Fig. 3). Note that reported phloem transport speeds in this study corresponds to front speeds of the fastest particles, revealing the speed of a concentration profile which might be distinctively different from group speed. While isotope tracing in the past was mostly restricted to time of first arrival and phloem transport speed, we here estimate carbon partitioning into surrounding tissues and the atmosphere using compartmental modelling (see also [9, 19, 37]) to readily address dynamics in axial carbohydrate transport and radial exchange in Arabidopsis.

A limitation of the compartmental modelling approach applied here is that PET measurements provide only the total ^11^C activity per ROI, integrating tracer dynamics across multiple tissues without direct observation of the individual compartments represented in the model. As a consequence, exchange parameters describing unloading, retrieval, storage and efflux are inferred indirectly from the combined signal, rather than from compartment-specific measurements. Although model calibration, sensitivity and identifiability analyses indicated that these parameters were identifiable for most datasets, their interpretation should be understood as reflecting relative partitioning of recently fixed carbon rather than absolute fluxes. Parameter identifiability further depended on data quality, which could vary between scans due to differences in tracer uptake and transport. This influences signal-to-noise ratio, particularly in thinner shoots or under lower flow conditions. These constraints are inherent to non-invasive PET-based measurements at sub-millimeter resolution and emphasize that the model represents a simplified description of integrated phloem processes. Nevertheless, the consistent parameter convergence across independent ROI sets and shoot types supports the use of this framework to compare relative differences in phloem transport dynamics and carbon partitioning between organs and along the shoot axis.

A next step would be to image partitioning of recently fixed ^11^C-carbon at cellular scale. This would need spatially or physically/chemically resolved compartments which is however not possible with PET since the spatial resolution of this technique is physically constraint by the positron range to sub-millimeter scale. Hence, the current model estimates fraction rates. Spatially resolved data and volumetric measurements are required to assess if observed differences in fractionation across compartments result in differences in carbohydrate fluxes. For now, the mathematical description makes it possible to estimate overall dynamics in parameters linked to sink strength, carbohydrate storage and respiratory activity.

### Method demonstration in Arabidopsis inflorescences

Froelich et al. [17] measured phloem sap transport speeds of 60 to 100 µm s^−1^ in the roots of 3-week-old Arabidopsis plants, based on fluorescence recovery after photobleaching of phloem-mobile probes. With high-resolution PET and ^11^C-carbon labelling, it was found that this range stretches from 32 to 216 µm s^−1^ in the inflorescences, depending on the branching hierarchy. Bernardini et al. [6] reported phloem speeds of 28.3 ± 4.4 µm s^−1^ in wild-type Arabidopsis shoots having the same age as the plants in this study. Hereby radioactive ^14^CO_2_ labelling was used in combination with Bremsstrahlung radiation detection. Comparison with our results is, however, difficult because of different experimental labelling procedures. The Arabidopsis plants in our study were pulse labelled for 7 min with radioactive ^11^CO_2_ and the peak in activity was recorded around 65–70 min post labelling (Fig. 2C). In the experiment by Bernardini et al. [6] pulse labelling lasted 120 min, so that the peak observed in our study, could not have been observed with their experimental procedure.

We expect that increased light intensities may alter the absolute estimates of the phloem front speed. In primary shoots, a two-fold higher phloem front speed was detected compared to secondary shoots (Fig. 4B). This increased speed is likely driven by increased unloading of carbon in the primary shoot (Fig. 4C). It is not new that the primary shoot receives more sugars than secondary shoots. Apical dominance has been studied for a long time, and is in more recent years explained by the stronger sugar demand of the primary shoot that suppresses bud bursting and growth of lateral branches [33, 35]. Retrieval was identified as a required component in the compartmental model and was found to play a larger role in the primary shoot, providing independent, mathematical support for the existence of a leakage-retrieval mechanism [55]. Our compartmental model showed that the unloaded and retrieved fraction rate of recently fixed ^11^C-carbon is several magnitudes higher in primary than in secondary shoots (Fig. 4C, D). Although we do not have volumetric measurements to underpin these estimates, this indicates that unloading and retrieval are important factors in the dominant shoot.

## Conclusions

We established that high-resolution PET imaging allows for non-invasive, real-time detection of dynamic phloem transport in Arabidopsis stems as thin as 1.3-to-1.8-mm in diameter. This has only recently been possible due to commercial availability of the latest generation PET scanners that include monolithic PET detector crystals which allow PET imaging at submillimeter spatial resolution. By coupling these PET data to a compartmental model, not only phloem sap front speed was quantified, but also dynamics in radial carbon partitioning between leakage-retrieval, carbon storage and respiratory efflux were estimated.

The workflow on primary and secondary shoots illustrated that the pipeline delivers stable, interpretable transport parameters. In this case study, phloem sap front speed was higher in primary shoots, accompanied by stronger unloading and retrieval dynamics. With that, we present new insights and a novel approach that relies on the concerted effort of high-resolution PET scanning and compartmental modelling. Combination of these two techniques foster new possibilities to test theoretical frameworks of phloem transport dynamics and address biological questions related to underlying mechanisms of carbohydrate partitioning.

## Supplementary Information

Additional file 1. Linear mixed models and generalized linear mixed models of the effect of shoot (primary versus secondary) on phloem front speed and carbon partitioning parameters.

Additional file 2. Calculation of the average travel distance upon annihilation for positrons that originate in phloem tissue of cylindrical Arabidopsis shoots with diameter *d*.

## Data Availability

The data supporting the findings of this study are available within the paper and its supplementary materials published online. All other data is available from the corresponding author, KS, upon request.

## References

[1] Alexoff DL, Dewey SL, Vaska P, Krishnamoorthy S, Ferrieri R, Schueller M, et al. PET imaging of thin objects: measuring the effects of positron range and partial-volume averaging in the leaf of *Nicotiana tabacum*. Nucl Med Biol. 2011;38:191–200. 21315274 10.1016/j.nucmedbio.2010.08.004

[2] Barratt DHP, Kolling K, Graf A, Pike M, Calder G, Findlay K, et al. Callose synthase GSL7 is necessary for normal phloem transport and inflorescence growth in Arabidopsis. Plant Physiol. 2011;155:328–41. 21098675 10.1104/pp.110.166330 PMC3075753

[3] van Bel AJE, Hafke JB. Physiochemical determinants of phloem transport. In: Holbrook NM, Zwieniecki MA, editors. Vascular transport in plants. Burlington: Elsevier; 2005. p. 19–44.

[4] van Bel AJE, Musetti R. Sieve element biology provides leads for research on phytoplasma lifestyle in plant hosts. J Exp Bot. 2019;70:3737–55. 30972422 10.1093/jxb/erz172

[5] Berardini TZ, Reiser L, Li D, Mezheritsky Y, Muller R, Strait E, et al. The Arabidopsis information resource: making and mining the ‘gold standard’ annotated reference plant genome. Genesis. 2015;53:474–85. 26201819 10.1002/dvg.22877 PMC4545719

[6] Bernardini C, Santi S, Mian G, Levy A, Buoso S, Suh JH, et al. Increased susceptibility to Chrysanthemum Yellows phytoplasma infection in Atcals7ko plants is accompanied by enhanced expression of carbohydrate transporters. Planta. 2022;256:1–17. 35842878 10.1007/s00425-022-03954-8 PMC9288947

[7] Black MZ, Minchin PEH, Gould N, Patterson KJ, Clearwater MJ. Measurement of bremsstrahlung radiation for in vivo monitoring of 14C tracer distribution between fruit and roots of kiwifruit (*Actinidia arguta*) cuttings. Planta. 2012;236:1327–37. 22729822 10.1007/s00425-012-1685-z

[8] Brun R, Kühni M, Siegrist H, Gujer W, Reichert P. Practical identifiability of ASM2d parameters—systematic selection and tuning of parameter subsets. Water Res. 2002;36:4113–27. 12405420 10.1016/s0043-1354(02)00104-5

[9] Bühler J, Huber G, Schmid F, Blümler P. Analytical model for long-distance tracer-transport in plants. J Theor Biol. 2011;270:70–9. 21056579 10.1016/j.jtbi.2010.11.005

[10] Cao J, Schneeberger K, Ossowski S, et al. Whole-genome sequencing of multiple *Arabidopsis thaliana* populations. Nat Genet. 2011;43:956–65. 21874002 10.1038/ng.911

[11] Conti M, Eriksson L. Physics of pure and non-pure positron emitters for PET: a review and a discussion. EJNMMI Phys. 2016. 10.1186/s40658-016-0144-5. 27271304 10.1186/s40658-016-0144-5 PMC4894854

[12] Delignette-Muller ML, Dutang C. fitdistrplus: an R package for fitting distributions. J Stat Softw. 2015;64:1–34.

[13] Dethloff F, Orf I, Kopka J. Rapid in situ 13C tracing of sucrose utilization in *Arabidopsis* sink and source leaves. Plant Methods. 2017;13:87. 29075313 10.1186/s13007-017-0239-6 PMC5648436

[14] Dong S, Zhang J, Beckles DM. A pivotal role for starch in the reconfiguration of (14)C-partitioning and allocation in *Arabidopsis thaliana* under short-term abiotic stress. Sci Rep. 2018;8:9314. 29915332 10.1038/s41598-018-27610-y PMC6006365

[15] Duan QY, Gupta VK, Sorooshian S. Shuffled complex evolution approach for effective and efficient global minimization. J Optim Theory Appl. 1993;76:501–21.

[16] Duan Q, Sorooshian S, Gupta VK. Optimal use of the SCE-UA global optimization method for calibrating watershed models. J Hydrol. 1994;158:265–84.

[17] Froelich DR, Mullendore DL, Jensen KH, Ross-Elliott TJ, Anstead JA, Thompson GA, et al. Phloem ultrastructure and pressure flow: sieve-element-occlusion-related agglomerations do not affect translocation. Plant Cell. 2011;23:4428–45. 22198148 10.1105/tpc.111.093179 PMC3269875

[18] Geiger DR, Swanson CA. Evaluation of selected parameters in a sugar beet translocation system. Plant Physiol. 1965;40:942–7. 16656179 10.1104/pp.40.5.942 PMC550410

[19] Hubeau M, Mincke J, Vanhove C, Courtyn J, Vandenberghe S, Steppe K. Plant-PET to investigate phloem vulnerability to drought in *Populus tremula* under changing climate regimes. Tree Physiol. 2019;39:211–21. 30597097 10.1093/treephys/tpy131

[20] Hubeau M, Steppe K. Plant-PET scans. In vivo mapping of xylem and phloem functioning. Trends Plant Sci. 2015;20:676–85. 26440436 10.1016/j.tplants.2015.07.008

[21] Jaeger CH, Goeschl JD, Magnuson CE, Fares Y, Strain BR. Short-term responses of phloem transport to mechanical perturbation. Physiol Plant. 1988;72:588–94.

[22] Jodal L, Le Loirec C, Champion C. Positron range in PET imaging: an alternative approach for assessing and correcting the blurring. Phys Med Biol. 2012;57:3931–43. 22643300 10.1088/0031-9155/57/12/3931

[23] Karve AA, Alexoff D, Kim D, Schueller MJ, Ferrieri RA, Babst BA. In vivo quantitative imaging of photoassimilate transport dynamics and allocation in large plants using a commercial positron emission tomography (PET) scanner. BMC Plant Biol. 2015;15:273. 26552889 10.1186/s12870-015-0658-3 PMC4640171

[24] Kaul M, Surti S, Karp JS. Combining surface treatments with shallow slots to improve the spatial resolution performance of continuous, thick LYSO detectors for PET. IEEE Trans Nucl Sci. 2013;60:44–52. 24077642 10.1109/TNS.2013.2240315 PMC3783277

[25] Knoblauch M, Vendrell M, de Leau E, Paterlini A, Knox K, Ross-Elliot T, et al. Multispectral phloem-mobile probes: properties and applications. Plant Physiol. 2015;167:1211–20. 25653316 10.1104/pp.114.255414 PMC4378168

[26] Knox K, Paterlini A, Thomson S, Oparka K. The coumarin glucoside, esculin, reveals rapid changes in phloem-transport velocity in response to environmental cues. Plant Physiol. 2018;178:795–807. 30111635 10.1104/pp.18.00574 PMC6181028

[27] Köckenberger W, Pope JM, Xia Y, Jeffrey KR, Komor E, Callaghan PT. A non-invasive measurement of phloem and xylem water flow in castor bean seedlings by nuclear magnetic resonance microimaging. Planta. 1997;201:53–63.

[28] Kolling K, Müller A, Flutsch P, Zeeman SC. A device for single leaf labelling with CO2 isotopes to study carbon allocation and partitioning in *Arabidopsis thaliana*. Plant Methods. 2013;9:1–12. 24252607 10.1186/1746-4811-9-45 PMC4177546

[29] Kölling K, Thalmann M, Müller A, Jenny C, Zeeman SC. Carbon partitioning in *Arabidopsis thaliana* is a dynamic process controlled by the plants metabolic status and its circadian clock. Plant Cell Environ. 2015;38:1965–79. 25651812 10.1111/pce.12512 PMC4671261

[30] Krishnamoorthy S, Blankemeyer E, Mollet P, Surti S, Van Holen R, Karp JS. Performance evaluation of the MOLECUBES β-CUBE - a high spatial resolution and high sensitivity small animal PET scanner utilizing monolithic LYSO scintillation detectors. Phys Med Biol. 2018;63:0–12. 10.1088/1361-6560/aacec3 PMC6145835 29938684

[31] Kutta W. Beitrag zur näherungsweisen Integration totaler Differentialgleichungen. Z Math Phys. 1901;46:435–53.

[32] Landais P, Finn R. On-line preparation of 11C carbon dioxide from 11C methane. Int J Radiat Appl Instrum A Appl Radiat Isot. 1989;40:265–6. 10.1016/0883-2889(89)90161-5 2541111

[33] Leyser O. The fall and rise of apical dominance. Curr Opin Genet Dev. 2005;15:468–71. 15964756 10.1016/j.gde.2005.06.010

[34] Loening AM, Gambhir SS. AMIDE: a free software tool for multimodality medical image analysis. Mol Imag. 2003;2:131–7. 10.1162/15353500200303133 14649056

[35] Mason MG, Ross JJ, Babst BA, Wienclaw BN, Beveridge CA. Sugar demand, not auxin, is the initial regulator of apical dominance. Proc Natl Acad Sci. 2014;111:6092–7. 24711430 10.1073/pnas.1322045111 PMC4000805

[36] Minchin PEH, Thorpe MR. Using the short-lived isotope 11C in mechanistic studies of photosynthate transport. Funct Plant Biol. 2003;30:831–41. 32689068 10.1071/FP03008

[37] Mincke J, Courtyn J, Vanhove C, Vandenberghe S, Steppe K. Studying in vivo dynamics of xylem-transported 11CO2 using positron emission tomography. Tree Physiol. 2020;40:1058–70. 32333788 10.1093/treephys/tpaa048

[38] Mincke J, Courtyn J, Vanhove C, Vandenberghe S, Steppe K. Guide to Plant-PET imaging using 11CO2. Front Plant Sci. 2021;12:1–18. 10.3389/fpls.2021.602550 PMC8206809 34149742

[39] Moorby AJ, Jarman PD. The use of compartmental analysis in the study of the movement of carbon through leaves. Planta. 1975;122:155–68. 24435965 10.1007/BF00388655

[40] Münch E. Die Stoffbewegingen in der Pflanze. Jena: G. Fischer; 1930.

[41] Nakanishi TM. What you can see by developing real-time radioisotope imaging system for plants: from water to element and CO2 gas imaging. J Radioanal Nucl Chem. 2018;318:1689–95. 30546186 10.1007/s10967-018-6324-0 PMC6267115

[42] Patrick JW. Fundamentals of Phloem Transport Physiology. In: Thompson GA, van Bel AJE, editors. Phloem: molecular cell biology, systemic communication, biotic interactions. Oxford: Wiley; 2013. p. 30–59.

[43] De Pauw DJW, Steppe K, De Baets B. Identifiability analysis and improvement of a tree water flow and storage model. Math Biosci. 2008;211:314–32. 17936856 10.1016/j.mbs.2007.08.007

[44] Pickard WF, Minchin PE. Nature of the short-term inhibition of stem translocation produced by abrupt stimuli. Aust J Plant Physiol. 1992;19:471–80.

[45] Pigliucci M. Ecology and evolutionary biology of *Arabidopsis*. Arabidopsis Book. 2002;1:e0003–e0003. 22303188 10.1199/tab.0003 PMC3243336

[46] Pinheiro J, Bates D, DebRoy S, Sarkar D, Team RC. Linear and nonlinear mixed effects models. 2018.

[47] R-Core-Team. R: a language and environment for statistical computing. Vienna: R Foundation for Statistical Computing; 2017.

[48] Rédei GP. A heuristic glance at the past of Arabidopsis genetics. Methods Arabid Res. 1992;1:1–15.

[49] Runge C. Über die numerische Auflösung von Differentialgleichungen. Math Ann. 1895;46:167–78.

[50] Saha GB. Basics of PET imaging: physics, chemistry, and regulations. New York: Springer; 2016.

[51] Schaart DR, Van Dam HT, Seifert S, Vinke R, Dendooven P, Löhner H, et al. A novel, SiPM-array-based, monolithic scintillator detector for PET. Phys Med Biol. 2009;54:3501–12. 19443953 10.1088/0031-9155/54/11/015

[52] De Schepper V, De Swaef T, Bauweraerts I, Steppe K. Phloem transport: a review of mechanisms and controls. J Exp Bot. 2013;64:4839–50. 24106290 10.1093/jxb/ert302

[53] Sugita R, Kobayashi NI, Hirose A, Saito T, Iwata R, Tanoi K, et al. Visualization of uptake of mineral elements and the dynamics of photosynthates in Arabidopsis by a newly developed real-time radioisotope imaging system (RRIS). Plant Cell Physiol. 2016;57:743–53. 27016100 10.1093/pcp/pcw056 PMC4836453

[54] Thorpe MR, Minchin PEH. In vivo veritas: radiotracers in studies of phloem transport of carbohydrate. In: Liesche J, editor. Phloem: methods and protocols. New York: Humana Press; 2019. p. 177–94. 10.1007/978-1-4939-9562-2_15 31197796

[55] Thorpe M, Minchin P, Gould N, McQueen J. The stem apoplast: a potential communication channel in plant growth regulation. Vasc Transp Plants. 2005. 10.1016/B978-012088457-5/50012-5.

[57] Turgeon R. The puzzle of phloem pressure. Plant Physiol. 2010. 10.1104/pp.110.161679. 20921188 10.1104/pp.110.161679 PMC2949042

[58] Turgeon R, Wolf S. Phloem transport: cellular pathways and molecular trafficking. Annu Rev Plant Biol. 2009. 10.1146/annurev.arplant.043008.092045. 19025382 10.1146/annurev.arplant.043008.092045

[59] Wickham H. ggplot2: elegant graphics for data analysis. New York: Cham; 2016.

[60] Wipf D, Pfister C, Mounier A, Leborgne-Castel N, Frommer WB, Courty P-E. Identification of putative interactors of Arabidopsis sugar transporters. Trends Plant Sci. 2021;26:13–22. 33071187 10.1016/j.tplants.2020.09.009

[61] Zeileis A, Hothorn T. Diagnostic checking in regression relationships. R News. 2002;2:7–10.

