# Supplementary material for: Investigating phloem transport dynamics in Arabidopsis through compartmental modelling of positron emission tomography data
Source: Plant Methods. 2026 Mar 30;22:47. doi: 10.1186/s13007-026-01525-6 (PMC13154432; doi:10.1186/s13007-026-01525-6)
Supplement: Supplementary file 1 — Additional file 1. Linear mixed models and generalized linear mixed models of the effect of shoot (primary versus secondary) on phloem front speed and carbon partitioning parameters. [file 13007_2026_1525_MOESM1_ESM.pdf]

Supplemental Data 1

Linear mixed models and generalized linear mixed models of the shoot effect (primary versus secondary shoot) on phloem front speed and ^{11}C partitioning parameters.

1. Phloem front speed

Likelihood ratio test

```
Model 1: v_ums ~ branch
Model 2: v_ums ~ 1
  #Df  LogLik Df  Chisq Pr(>Chisq)
1   4 -145.00
2   3 -160.75 -1 31.484  2.011e-08 ***
```

Linear mixed-effects model fit by REML

```
  Data: x
       AIC      BIC    logLik
  298.0068 303.6116 -145.0034

Random effects:
 Formula: ~1 | plant
        (Intercept) Residual
StdDev:    18.64617 25.52325

Fixed effects:  v_ums ~ branch
                Value Std.Error DF  t-value p-value
(Intercept) 127.55694 11.297550 27 11.29067       0
branchside  -56.89418  9.082245 27 -6.26433       0
 Correlation:
           (Intr)
branchside -0.397

Standardized Within-Group Residuals:
       Min         Q1        Med         Q3        Max
-1.3790459 -0.7346778 -0.1145266  0.4112256  2.7672883

Number of Observations: 32
Number of Groups: 4
```

2. Fraction a_{12}

Likelihood ratio test

```
Model 1: a12 ~ branch + (1 | plant)
Model 2: a12 ~ 1
  #Df LogLik Df  Chisq Pr(>Chisq)
1   3 34.820
2   2 25.003 -1 19.634   9.38e-06 ***
```

Generalized mixed-effects model

```
glm(formula = a12 ~ branch + (1 | plant), family = Gamma, data = x)

Deviance Residuals:
    Min       1Q   Median       3Q      Max
-1.4340  -0.7301  -0.1591   0.4358   1.1447


              Estimate Std. Error t value Pr(>|t|)
(Intercept)     3.7522     0.6836   5.489 5.86e-06 ***
branchside     10.1908     2.6306   3.874 0.000539 ***

(Dispersion parameter for Gamma family taken to be 0.5310659)
```

```
Null deviance: 30.501  on 31  degrees of freedom
Residual deviance: 17.606  on 30  degrees of freedom
AIC: -63.641

Number of Fisher Scoring iterations: 6
```

3. Fraction a_{21}

Likelihood ratio test

```
Model 1: a21 ~ branch + (1 | plant)
Model 2: a21 ~ 1
  #Df LogLik Df Chisq Pr(>Chisq)
1   3 46.833
2   2 36.993 -1 19.68  9.156e-06 ***
```

Generalized mixed-effects model

```
glm(formula = a21 ~ branch + (1 | plant), family = Gamma, data = x)

Deviance Residuals:
    Min       1Q   Median       3Q      Max
-1.4015  -0.7301  -0.1591   0.4353   1.1447


              Estimate Std. Error t value Pr(>|t|)
(Intercept)     5.4570     0.9943   5.488 5.87e-06 ***
branchside     14.7962     3.8220   3.871 0.000543 ***

(Dispersion parameter for Gamma family taken to be 0.5312368)

Null deviance: 30.384  on 31  degrees of freedom
Residual deviance: 17.512  on 30  degrees of freedom
AIC: -87.666

Number of Fisher Scoring iterations: 6
```

4. Fraction b

Likelihood ratio test

```
Model 1: b ~ branch
Model 2: b ~ 1
  #Df LogLik Df  Chisq Pr(>Chisq)
1   4 20.121
2   3 13.737 -1 12.768  0.0003526 ***
```

Linear mixed-effects model fit by REML

```
  Data: x
        AIC       BIC   logLik
  -32.24234 -26.63755 20.12117

Random effects:
 Formula: ~1 | plant
        (Intercept)  Residual
StdDev:  0.09133307 0.1021342

Fixed effects:  b ~ branch
                 Value  Std.Error DF  t-value p-value
(Intercept) 0.07833529 0.05232012 27 1.497231  0.1459
branchside  0.17818016 0.03635978 27 4.900475  0.0000
 Correlation:
           (Intr)
branchside -0.343

Standardized Within-Group Residuals:
```

```
        Min          Q1         Med          Q3         Max
-2.07076467 -0.64387716  0.05955859  0.50049087  2.06095008

Number of Observations: 32
Number of Groups: 4
```

5. Fraction c

Likelihood ratio test

```
Model 1: c ~ branch + (1 | plant)
Model 2: c ~ 1
  #Df LogLik Df  Chisq Pr(>Chisq)
1   3 7.0855
2   2 6.5394 -1 1.0922      0.296
```

Generalized mixed-effects model

```
glm(formula = c ~ branch + (1 | plant), family = Gamma, data = x)

Deviance Residuals:
     Min        1Q    Median        3Q       Max
-1.48227  -0.79703  -0.00861   0.48775   0.88225

              Estimate Std. Error t value Pr(>|t|)
(Intercept)     2.6144     0.4048   6.459 3.89e-07 ***
branchside      0.7586     0.6607   1.148     0.26

(Dispersion parameter for Gamma family taken to be 0.3835096)

Null deviance: 16.701  on 31  degrees of freedom
Residual deviance: 16.183  on 30  degrees of freedom
AIC: -8.1709

Number of Fisher Scoring iterations: 5
```
