# Supplementary material for: Investigating phloem transport dynamics in Arabidopsis through compartmental modelling of positron emission tomography data
Source: Plant Methods. 2026 Mar 30;22:47. doi: 10.1186/s13007-026-01525-6 (PMC13154432; doi:10.1186/s13007-026-01525-6)
Supplement: Supplementary file 2 — Additional file 2. Calculation of the average travel distance upon annihilation for positrons that originate in phloem tissue of cylindrical Arabidopsis shoots with diameter d. [file 13007_2026_1525_MOESM2_ESM.pdf]

Supplemental Data 2

Positron range is the distance that the positron travels through an object to lose an excess of kinetic energy before annihilation takes place. The mean distance between a decaying nucleus and the site of annihilation (i.e., mean positron range) for carbon-11 (^{11}C) is 1.2 mm and can amount to maximum 4.2 mm (Conti & Eriksson 2016). If the positron originates close to the tissue surface, the possibility exists that the positron escapes the tissue and travels in the air to annihilate at another surface.

This supplemental data describes the calculation of the annihilation probability for ^{11}C-positrons that originate in the phloem tissue of cylindrical Arabidopsis tissues having a diameter *d* and in which the centre of the phloem tissue is located at 0.09 **d* of the stem surface (Fig. S1) as determined by transverse Arabidopsis plant sections. To do this, the average travel distance of a positron within the stem is determined, which is then converted to the annihilation probability according to Jodal *et al.* (2012).

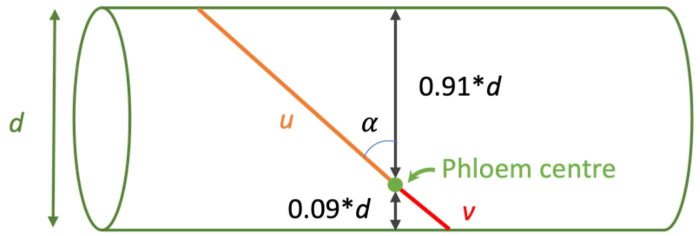

Figure S1. Schematic representation of a cylindrical Arabidopsis stem (with diameter d) in which the centre of the phloem tissue is located at *d**0.09 of the stem surface. A cross section of the stem under angle α has an elliptical shape that is characterised by a minor and major axis of length *d* and ($u + v$).

1. Calculating the average travel distance of an ^{11}C-positron in an Arabidopsis stem

The calculation of the average travel distance of an ^{11}C-positron was divided into two parts. First the 3D volume (within the Arabidopsis stem) was defined in which the positron can travel. Next the average travel distance is calculated within this 3D volume.

1.1 Defining the 3D volume that an ^{11}C-positron can travel in an Arabidopsis stem

A 3D volume can be generated by defining the cross section of the stem as a function of angle α. If $\alpha = k\pi$ (with *k* an integer), the cross section is a circle (with diameter *d*) but for all other angles it becomes an ellipse that is characterised by a minor and major axis of length *d* and ($u + v$) as displayed in Fig. S2. An elliptical cross section can be parameterised by $(x, y) = (a \cos\theta, b \sin\theta)$ with *a*, *b* the semi-major and semi-minor axes which equal $(u+v)/2$ and $d/2$, respectively. Note that only *u* and *v* depend on α. Due to symmetry, only one quadrant can be regarded so that $\alpha \in [0, \pi/2]$.

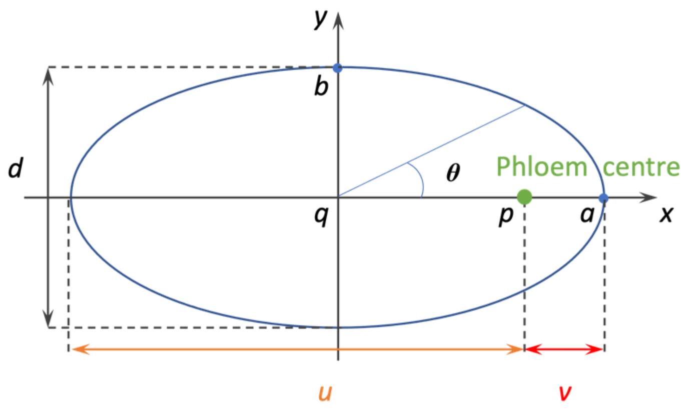

Figure S2. Schematic representation of the elliptical cross section defined by α. The ellipse is characterised (x, y) = (a cosθ, b sinθ) with semi-major and semi-minor axis a and b, which corresponds to length d/2 and (u + v)/2. The phloem centre has coordinates (p, q).

Next α is determined for which the largest distance between the phloem centre and the surface of the stem becomes 4.2 mm. This largest distance is always defined by the major axis (i.e., u+v) of the cross section. Two values for α are found, $\cos^{-1}(0.91d/4.2)$ and $\cos^{-1}(0.09d/4.2)$, i.e., when u and v reach 4.2 mm, respectively. From these angles on their length is capped at 4.2 mm. In this way, a can be defined by three equations Eq. (1-3), depending on the thresholds for α .

For $\alpha \in [0, \cos^{-1}(0.91d/4.2)]$,

$$a = \frac{u+v}{2} = \frac{0.91d/\cos(\alpha) + 0.09d/\cos(\alpha)}{2} = \frac{d}{2\cos(\alpha)} \qquad (1)$$

For $\alpha \in [\cos^{-1}(0.91d/4.2), \cos^{-1}(0.09d/4.2)]$,

$$a = \frac{u+v}{2} = \frac{4.2 + 0.09d/\cos(\alpha)}{2} \qquad (2)$$

For $\alpha \in [\cos^{-1}(0.09d/4.2), \pi/2]$,

$$a = \frac{u+v}{2} = \frac{4.2+4.2}{2} = 4.2 \qquad (3)$$

Hereby, the 3D volume in which a positron can travel is characterised by elliptical cross sections that are defined by a (Eq. 1-3) and $b = d/2$.

1.2 Defining a function for the distance from the phloem centre point (*p*, *q*) to a point on an ellipse

Since the 3D volume is defined by a set of ellipses, the average travel distance for the total 3D volume can be divided into calculating the average travel distance for an ellipse. Unfortunately, no equation is available so that it will be derived in this section.

The square distance D between a point on an ellipse centred at the origin (0,0) and the phloem centre point (p, q) can be calculated according to Eq. (4).

$$D^2(\theta) = (p - a\cos\theta)^2 + (q - b\sin\theta)^2 \qquad (4)$$

The square function for the average distance $\dot{D}$ for n distances spread evenly over 2π is given by Eq. (2).

$$\dot{D}^2 = \frac{\sum_{k=0}^{n-1} D^2(\frac{2\pi k}{n})}{n} \quad (5)$$

Substituting Eq. (4) in Eq. (5) results in Eq. (6) by making use of trigonometric half angle identities and the fact that $\sum_{k=0}^{n-1} \cos\left(\frac{2k\pi}{n}\right)=0$, $\sum_{k=0}^{n-1} \sin\left(\frac{2k\pi}{n}\right)=0$, $\sum_{k=0}^{n-1} \cos\left(\frac{4k\pi}{n}\right)=0$ and $\sum_{k=0}^{n-1} \sin\left(\frac{4k\pi}{n}\right)=0$ because θ is evenly spaced over 2π.

$$\dot{D}^2 = \frac{n\left(p^2 + \frac{a^2}{2} + q^2 + \frac{b^2}{2}\right)}{n} = p^2 + \frac{a^2}{2} + q^2 + \frac{b^2}{2} \quad (6)$$

Variables *a* and *b* were identified in the previous section so that only coordinates (*p*, *q*) of the phloem centre needs to be found. Since the phloem centre is located on the x axis, *q* always equals zero (Fig. S2). Variable *p* can be found by subtracting *v* from *a*. Hereby, a is given by Eq. (1-3) whereas $v = 0.09\, d/\cos(\alpha)$ for $\alpha \in [0, \cos^{-1}(0.09d/4.2)]$ and 4.2 for $\alpha \in [\cos^{-1}(0.09d/4.2), \pi/2]$.

Eventually, three separate functions $\dot{D}^2(\alpha)$ are found using the same division for α as in section 1.1. The square root of these functions defines the average travel distance for each range of α. The average travel distance can be found by integrating these square root equations over their respective α-range, summing these values up and divide by π/2. The only unknown in this sum is the plant's diameter *d*.

2. Converting the average travel distance to annihilation probability

When the average travel distance is calculated according to section 1. the corresponding annihilation probability can be found through the empirical probability curves of Jodal *et al.* (2012). These curves describe the cumulative annihilation probability distribution of positrons in function of travel distance and are different for each isotope. Fig. S3 shows the annihilation probability distribution for ^{11}C-positrons.

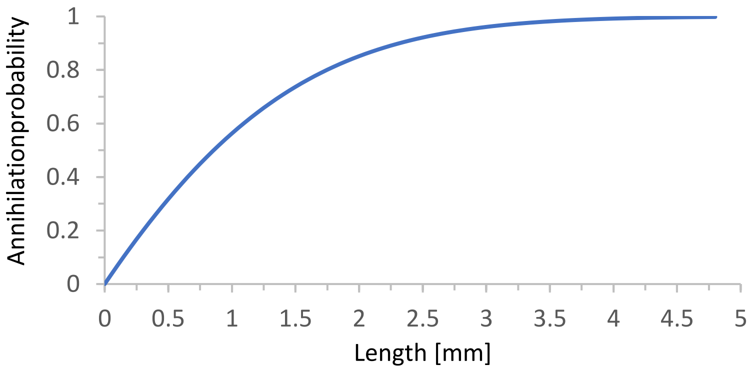

Figure S3. Annihilation probability distribution function of ^{11}C-positrons

Examples

For an Arabidopsis stem with a diameter d = 2 mm, the average travel distance of a positron that originated in the phloem centre is 1.7 mm which corresponds to an annihilation probability of 0.79. Therefore, it can be concluded that 79% of the positrons will annihilate in the tissue.

References

Conti, M. & L. Eriksson. (2016). Physics of pure and non-pure positron emitters for PET: A review and a discussion. *EJNMMI Phys.*, 3.

Jodal, L., C. Le Loirec & C. Champion. (2012). Positron range in PET imaging: An alternative approach for assessing and correcting the blurring. *Phys. Med. Biol.*, 57, 3931–3943.
